# Household living conditions and individual behaviours associated with malaria risk: a community-based survey in the Limpopo River Valley, 2020, South Africa

**DOI:** 10.1186/s12936-023-04585-4

**Published:** 2023-05-15

**Authors:** Sean M. Patrick, Marc-Karim Bendiane, Taneshka Kruger, Bernice N. Harris, Megan A. Riddin, Helene Trehard, Christiaan de Jager, Riana Bornman, Jean Gaudart

**Affiliations:** 1 UP Institute for Sustainable Malaria Control & MRC Collaborating Centre for Malaria Research, School of Health Systems and Public Health, University of Pretoria, Private Bag X20, Hatfield, Pretoria, 0028 South Africa; 2 Economics & Social Sciences Applied to Health & Medical Information Processing, Aix Marseille University, INSERM, IRD, ISSPAM, SESSTIM, 13005 Marseille, France; 3 Aix Marseille University, INSERM, IRD, APHM, ISSPAM, SESSTIM, UMR1252, Hospital La Timone, BioSTIC, Biostatistics & ICT, 13005 Marseille, France

**Keywords:** Malaria, Limpopo, South Africa, Border, Community-based survey, Health Behaviors, Housing conditions

## Abstract

**Background:**

Over the past decade, implementation of multiple malaria control strategies in most countries has largely contributed to advance the global malaria elimination agenda. Nevertheless, in some regions, seasonal epidemics may adversely affect the health of local populations. In South Africa, *Plasmodium falciparum* malaria is still present, with the Vhembe District experiencing an incidence rate of 3.79 cases/1000 person-years in 2018, particularly in the Limpopo River Valley, bordering Zimbabwe. To elucidate the complexity of the mechanisms involved in local regular malaria outbreaks, a community-based survey was implemented in 2020 that focused on the relationship between housing conditions and malaria risky behaviours.

**Methods:**

The community-based cross-sectional survey was conducted among the population of three study sites in the Vhembe District, which were selected based on malaria incidence rate, social and health characteristics of inhabitants. The household survey used a random sampling strategy, where data were collected through face-to-face questionnaires and field notes; to described the housing conditions (housing questionnaire), and focus on individual behaviours of household members. Statistical analyses were performed combining hierarchical classifications and logistic regressions.

**Results:**

In this study, 398 households were described, covering a population of 1681 inhabitants of all ages, and 439 adults who participated in community-based survey. The analysis of situations at risk of malaria showed that the influence of contextual factors, particularly those defined by the type of habitat, was significant. Housing conditions and poor living environments were factors of malaria exposure and history, regardless of site of investigation, individual preventive behaviours and personal characteristics of inhabitants. Multivariate models showed that, considering all personal characteristics or behaviours of inhabitants, housing conditions such as overcrowding pressures were significantly associated with individual malaria risk.

**Conclusions:**

The results showed the overwhelming weight of social and contextual factors on risk situations. Considering the Fundamental Causes Theory, malaria control policies based on health behaviour prevention, should reinforce access to care or promoting health education actions. Overarching economic development interventions in targeted geographical areas and populations have to be implemented, so that malaria control and elimination strategies can be efficiently and effectively managed.

## Background

Scaling up of malaria control strategies has achieved a remarkable reduction in the burden of malaria worldwide [1–4]. However, only 25 countries among 109 reached the pre-elimination/elimination stage [4]. Despite worldwide strategies to control malaria in middle and low incomes countries, ongoing epidemic dynamics are currently observed in certain malaria-endemic area. In South Africa, *Plasmodium falciparum* accounts for the majority of malaria cases and the main vector, *Anopheles arabiensis*, seems to be gradually giving way to new species [5]. Malaria remains a concern in three South African provinces bordering Zimbabwe and Mozambique, namely the Limpopo, Mpumalanga and KwaZulu-Natal provinces, where annual resurgence of malaria cases is observed [6]. However, even in the districts closest to the border, where most of the malaria burden is observed, malaria transmission intensity is highly heterogeneous and associated with low socio-economic status [7].

Currently, in South Africa, imported malaria cases are estimated to represent 47% of the total reported cases [8]. In South Africa, the Vhembe District Municipality (VDM), located in the north of Limpopo Province, has the highest (and increasing) number of malaria cases and deaths (3.79 cases per 1000 inhabitants-year during the 2017–2018 transmission season) compared to the other two malaria endemic provinces [9]. Furthermore, concerning, the Eastern Limpopo border in 2014, last available official data show more than 60% of the national cases were reported in the VDM. From 1998 to 2007, 65.6% of cases reported in Limpopo were located in VDM. More specifically, Mutale, which is one of the four municipalities in VDM, reported a total 15 739 cases from January 1998 to May 2017, this accounts for 27.1% of the total cases reported in VDM during the same period [10]. The VDM District has remained relatively unchanged compared to other provinces. Over the period 2002 to 2019, the estimated population growth of the VDM was 260 000 to a current estimate of 1.4 million people, thereby increasing the number of people at risk. The poverty headcount in Limpopo increased from 10.1% in 2011 to 11.5% in 2016. Among the 5.8 million residents in the Limpopo province, the 2016 census estimated that 389,151 residents moved to the province from elsewhere [11].

A better understanding of persistence of malaria in this specific area of South Africa has become a public health challenge. Malaria control policies face the emergent need to adapt to global goals and targeted interventions reaching elimination. Single intervention approaches based on health behaviours and health education, such as repellents and bed net use have been used previously, however, malaria cases still remain high in endemic areas. More complex studies reported on the importance of measuring multilevel factors, such as parasite genetics, societal organization, individual behaviours and geographic context, associated in dynamics of malaria in other countries. In literature, these multilevel factors are investigated using mixed medializations developed by the society-behaviour-biology approach based on Glass and McAtee’s works. The integration of natural and behavioural influences aid in modelling how and why individual health could be influenced at different levels and may change across the life course [12].

Indeed, if the health of populations is mainly the result of human activities including both individual behaviour and social organization, explaining the persistence of malaria requires to consider all the dimensions of this phenomenon. This study focused on individual health behaviours, i.e. behaviours that have deleterious or protective consequences on individual health, and this complexity forces us to study human exposure to malaria risks at three interconnected levels of people living conditions: (i) characteristics and changes in nature and climate; (ii) social contextual factors such as societal organization, cultural environment and access to health-related goods, and (iii) individual intention associated behaviours.

In line with this approach, this community-based survey aimed to analyse the association of these factors with having at least one malaria episode and relate these findings to housing condition and health behaviour, among inhabitants in a malaria endemic area in the east Limpopo River Valley.

## Methods

### Design

A community-based cross-sectional survey combining both household and individual data collection has been conducted at three selected sites of the east Limpopo River Valley (LPV) using face-to-face questionnaires and *in-situ* field notes.

Due to unavailability of current census data, area frame sampling was based on mapping geographical units. This was augmented with the aerial photographs to identify all household units’ location and ensure optimal coverage of the targeted population. Bende Mutale, Nkotswi and Doreen farms were chosen as investigation sites due to the particular high malaria incidences and known main characteristics of inhabitants, which include seasonal border mobility of workers or migrants. Bende Mutale and Nkotswi are two villages, with a majority of local population, along the Mutele River. Doreen Farms encompasses a number of farms 10 km along the Nzhelele River east of where the Doreen road crosses the R508 (10.3 km from the Tshipise Forever Resort and 22.1 km from the Musina Nature Reserve).

Common residence rules (*de jure* rules) defined household unit as group of inhabitants usually living in the same housing unit, which is a separate living quarter. All housing units have been systematically visited during the two field investigations. Data collections were planned in two different time periods to maximize contact with mobile and/or hidden population groups such as migrant (border) workers. A high contact rate mechanically increases the response rate of all eligible household members. Low-level equipment, such as landline or mobile phones, coupled with the lack of census data, prohibited the use of alternative multiple frame sampling processes.

### Data collection

During 2019, two data sets were collected and the household data was collected through face-to-face questionnaire with the head of household (older adult present during the investigation). With help of field notes (*in-situ* observations), additional information captured included structures of families living in the housing unit, incomes and goods, housing conditions, the bedding practices of all members and malaria history of household members. The individual level data was also collected through another face to face questionnaire on Knowledge, Attitudes and Practices toward malaria, administered to all household members aged over 18 years and *in-situ* during the inquiries visit. Having at least one malaria episode during the previous years (from January 2018) was used as the response variable.

### Statistical analyses

To assess household social profiles, household unit data was analysed by unsupervised classification using the hierarchical ascendant classification on the multiple correspondence analysis results. The study determined the most homogeneous household characteristics groups, using v.test to describe how each variable influences each category. The household resulting classification was then analysed using the following approach, similarly to other variables [13].

The description of the household profiles and KAP characteristics has been made after applying a weighting procedure, to ensure that the data were representative of the local population in relation to age and gender. Applied weights have been calculated as regard as Bende-Mutale inhabitants’ characteristics drawn out national census in 2011. When comparing those having reported a malaria episode during the previous years (from January 2018) to those who did not among KAP respondents (22), all descriptive analyses have been also weighted as well as aetiologic ones.

Chi-squared tests and Student T-tests were used in univariate analyses. To identify the associated factors, multivariate logistic regression model was used. During the univariate analysis, eligible variables for the multivariate analysis was selected with both 20% significance threshold and supporting evidence from literature. However, considering the small size of the sample, candidate variables were selected avoiding interactions between covariates especially in case of qualitative collinearity (redundant variables controlling the same underlying factor).

A multivariate logistic regression was then performed, using a step-by-step procedure based on the Akaike Information Criteria. The classification procedure was performed using the R 4.0.0 software (R foundation for statistical computing, Vienna, Austria) with the {Factominer} package. Univariate and multivariate analysis were performed using the IBM SPSS Statistics for Windows, Version 27.0 software (IBM Corp. Armonk, NY). The final test results were interpreted applying a fixed threshold at α = 0.05.

## Results

### Survey location

**Fig. 1:**
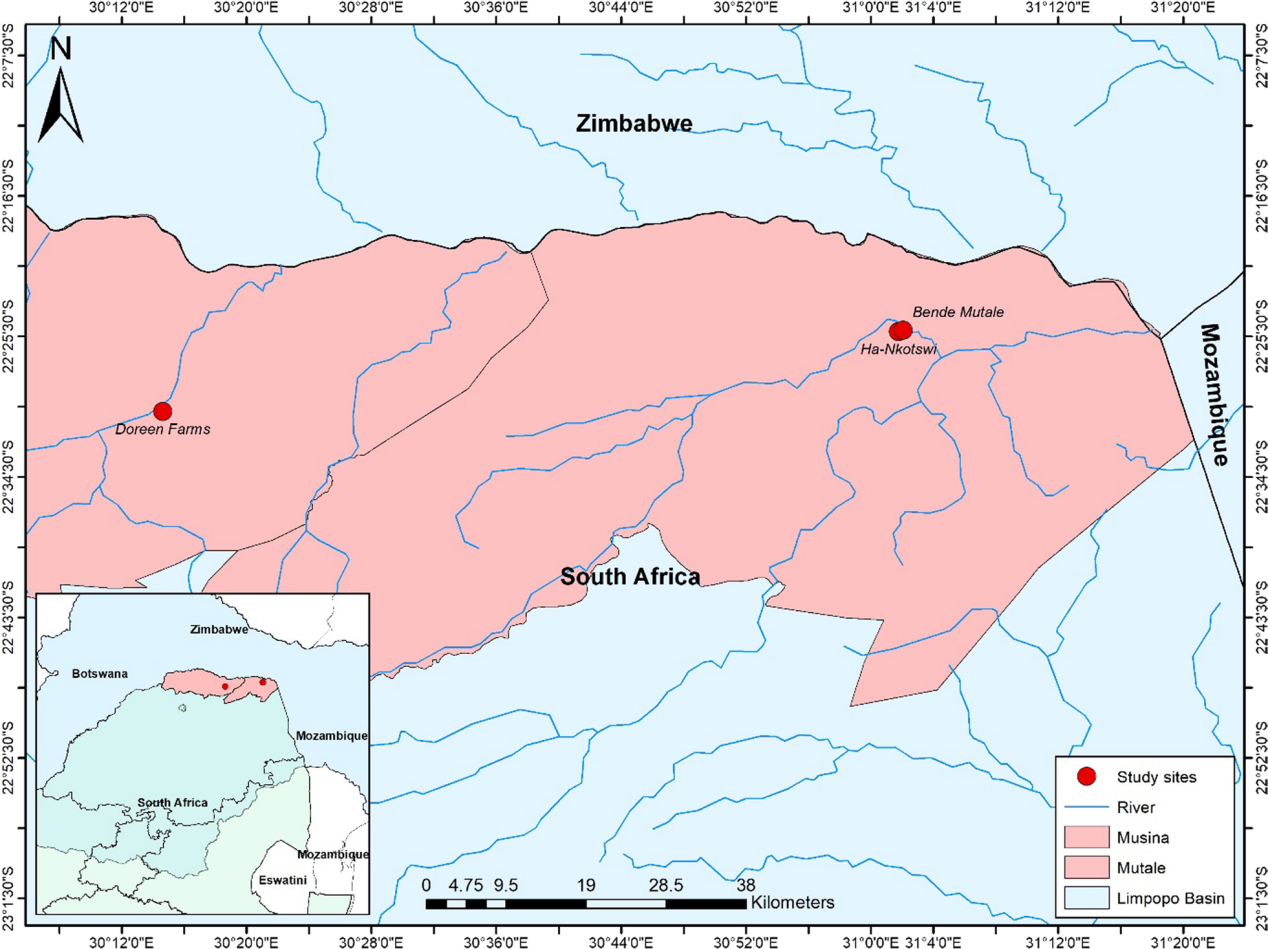
Limpopo River Valley, indicating study area [14]

The Vhembe Municipal District, Limpopo Province, is the area most heavily impacted by malaria in South Africa. The VDM borders Zimbabwe and Mozambique and is characterized by substantial trans-border movement of people, including temporary migrant workers. The Limpopo River valley has a high annual malaria incidence. The study sites we used for sampling were Bende Mutale, Ntokswi and the worker village at Doreen Farms (Fig. 1).

### Recruitment

During the two-field investigations administered during working hours, between August–December 2019, a total of 398 household units were investigated with 1,681 members. Among them, 949 were aged 18 years and older and eligible to participate in the KAP survey. Only 439 participated in the KAP data collection with a crude response rate of 46.3%, which ranged from 34.1% in Nkotswi to 60.3% to Doreen Farms (Table 1). When comparing eligible household members respondents to non-respondents, women were significantly more prone to participate in the survey than men (57.6% versus 31.5%; p < 0.001). Mean age of respondents were also significantly older than non-respondents (39.1 [standard error (se) = 15.7] versus 35.7 [se = 15.8]; p < 0.001).

**Table 1:** Recruitment and response rates by selected sites (South Africa, n = 1681)

Site	Bende-Mutale	Doreen farms	Nkotswi
HH_units (n)	200	152	46
HH members (n)	952	508	221
Eligible aged ≥ 18yo (n)	510	307	132
Respondents (n)	207	187	45
Crude response rate (n)	*40.6%*	*60.9%*	*34.1%*

### Household social profiles

Classification was performed including data collected by the household questionnaire which included household members’ and housing unit’s main characteristics. Among the 1681 targeted by the survey, 12 were excluded during the classification process and 1 669 household members were grouped in three main classes: class 1 = 1202, class 2 = 109, and class 3 = 358. When comparing these classes, with regard to social characteristics of household, housing conditions and malaria history (Table 2), three specific profiles can be defined. Major household characteristics collected became operational indicators pragmatically tailored to build social profiles contrasted that consider the specificity of the local population (poor area with lot of seasonal workers). Two major axes organize those indicators related the economic status that ranges from poor to less poor, and the family structure composing the household opposing workers and/or migrants to traditional family units (parent with children): Level of richest of the household unit was indicated by level of incomes, access to goods, housing equipment and quality of the house construction or proxy such as education level. Worker/migrant or family unit composing household was defined mainly through structure of the household (age, gender, size), but also by source and level of incomes or housing equipment basically nature of permanent goods.

**Table 2:** Household social profiles (South Africa, n = 1669)

	Profile 1	Profile 2	Profile 3	2 vs 1 P values	3 vs 1 P values
Household main social characteristics					
Family structure					
≥ One member aged under 12 years (%)	81.3	28.4	81.2	< 0.001	0.954
Single parent (%)	21.0	3.7	15.6	< 0.001	0.026
Single men (%)	0.0	24.8	0.0	< 0.001	–-
< 4 Household members (%)	16.3	64.2	21.2	< 0.001	0.050
Highest Education level among Housedhold members					
≥ One member at College/University (%)	1.3	0.9	26.3	0.715	< 0.001
Household unit income					
Monthly income > 2000 rds (%)	36.1	53.2	45.8	< 0.001	< 0.001
Household unit goods					
Car (%)	19.7	14.7	32.1	0.212	< 0.001
Electricity (%)	73.4	49.5	78.5	< 0.001	0.051
Electricity used for cooking (%)	13.5	11.0	24.9	0.467	< 0.001
TV and/or Radio (%)	73.4	49.5	78.5	0.014	< 0.001
Cell phone (%)	84.1	71.6	96.4	0.001	< 0.001
Animals (%)	89.5	55.0	93.3	< 0.001	0.033
Permanent Animals enclosure (%)	27.6	12.8	35.5	< 0.001	0.004
Permanent Water Barrell (%)	14.9	4.9	20.9	0.013	0.017
Housing conditions					
Size of the house					
Toilette outside home (%)	47.4	33.0	64.5	0.004	< 0.001
> 2 sleeping rooms (%)	32.90	25.00	51.8		< 0.001
Occupancy rate of sleeping rooms (%)	2.9 (1.6)	1.7(8.2)	2.1 (1.1)	< 0.001	< 0.001
Quality of the building					
Gap observed in doors (%)	64.3	56.9	69.6	0.123	0.067
Gap observed in the roof (%)	87.6	80.7	77.7	0.041	< 0.001
Gap observed in windows (%)	64.2	76.1	70.1	0.012	0.040
Wall in mud bricks (%)	30.2	39.4	49.2	0.046	< 0.001
Floor in cement (%)	81.9	75.2	93.0	0.089	< 0.001
Thatched roof (%)	27.2	16.5	14.5	0.015	< 0.001
Building in progress (%)	19.4	23.9	24.6	0.158	0.033
Malaria History of members					
Prevention					
House sprayed during the past year (%)	91.3	83.5	94.1	0.007	0.088
All household members using bed net (%)	0.0	10.1	52.0	< 0.001	< 0.001
Disease impact					
≥ One member got malaria during the five past years (%)	70.6	41.6	62.5	< 0.001	0.006

*Profile 1:* the first profile contained all members pooled in class 1, designated as relatively poor family (RPF). A household unit was composed by a significantly higher part of single-parent families (woman living with at least one child aged under 12 years) with several social vulnerability indicators such as education, incomes and available goods. Furthermore, housing conditions were more deleterious regarding the size of the house and the quality of the construction. Malaria exposure of the members was more important, considering bed net use and more strongly attested by impact on infection based of reporting of malaria episode during the past 5 years (Table 3).

**Table 3:** Associated factors with a recent malaria episode (South Africa, n = 439)

	Had declared at least one malaria episode from 2018				
	[1]* NO*	[2]* YES*	[1]* vs *[2]		
			*Univariate analysis*	*Multivariate analysis*	
	*n*_*w*_ = *372*	*n*_*w*_ = *67*	X^2^ p value	AOR (a)	CI 95% (b)
	*(100%)*	*(100%)*			
HHould Location and housing conditions profile					
Profiles					
Missing	2.2%	6.1%	0.017	–	–
[1] Low income family	54.8%	69.7%		2.619	1.152–5.956
[2] Migrant workers	17.7%	10.6%		1.339	0.438–4.099
[3] High income family [ref]	25.3%	13.6%		1	–
Villages					
Bende Mutale [ref]	50.5%	29.9%	< 0.001	1	
Doreen Farms	43.5%	38.8%		2.302	1.111–4.774
Nkotswi	5.9%	31.3%		8.638	3.797–19.653
Demographic characteristics					
Sex					
Woman [ref]	58.9%	51.5%	0.265	1	
Man	41.1%	48.5%		1.846	1.006–3.388
Age					
< 40 year [ref]	63.8%	57.4%	0.465		
40–64 year	23.9%	30.9%			
> 64 year	12.3%	11.8%			
Attitudes toward Malaria					
Fear of Malaria					
Yes	77.2%	86.6%	0.084	ns	–
No [ref]	22.8%	13.4%			
Opinions on life conditions					
Housing condition is as major problem					
Yes	11.8%	1.5%	0.010	0.115	0.013–0.995
No [ref]	88.2%	98.5%		1	–
Location is as major problem					
Yes	43.8%	44.8%	0.195	ns	–
No [ref]	56.2%	55.2%			
Night-times activities					
OUTSIDE at night					
Yes	87.1%	79.1%	0.084	ns	–
No [ref]	12.9%	20.9%			
Knowledge on Malaria					
Heard on Malaria					
Yes	84.8%	90.9%	0.159	ns	–
No[ref]	15.2%	9.1%			
Malaria at-risk behaviours					
OUTSIDE at night					
Yes	88.3%	81.8%	0.116	ns	–
No [ref]	11.7%	18.2%			
Prevention when outside at night					
Yes	37.9%	27.6%	0.088	ns	–
No [ref]	62.1%	72.4%			
Night-times health behaviours					
Use spray outside at night					
Yes	9.4%	17.9%	0.038	ns	–
No [ref]	90.6%	82.1%			
Sleep outside					
Yes	57.8%	70.1%	0.058	1.813	0.915–3.594
No [ref]	42.2%	29.9%		1	–

Adjusted Odds Ratios; (b) Confidence Interval

*Profile 2*: the second class, named as worker/migrant profile (WMP), was significantly composed by more single men and small family units than the two other profiles. Despite a high level of incomes, members have a low level of education and low access to permanent goods, such as electricity, TV/radio, animal, or house equipment (water barrel, electricity, animal enclosure). Housing conditions reflected family structure with small homes but low occupancy rate. Housing conditions were not deleterious compared to the first class but was deleterious compared to access to permanent goods. Concerning malaria prevention, use of bed nets were limited, but significantly fewer members declared having a malaria episodes during the past 5 years. Other specific elements confirmed the suitably of this profile regarding hidden characteristic or mobility. A large part of seasonal workers or migrants living in Doreen Farm refused to give nominal information (name and/or surname). Furthermore, more than one of five household members of profile 2, who declared having malaria episode, reported that they were not at Doreen Farm, but half were from other countries such as Zimbabwe. Field notes indicated that men’s clothes differ significantly in this group, as men wear long trousers, long sleeve and socks, which indicate regularly wearing working clothes.

*Profile 3:* The third profile differed significantly from the first based on all economic indicators, access of good and housing conditions. Malaria prevention was better known as well as impact of the disease. However, the family structure of the household unit was similar to those grouped under the first profile and, differs significantly from those labelled as profile 2. By contrast, this profile was named as Relatively Rich Family (RRF).

### Knowledge, attitudes and practices toward malaria

Concerning specific knowledge on malaria and malaria prevention, 83.9% of the survey respondents identified mosquitoes as the main vector of infection and for 56.2%, fever as the main symptom of the disease. However, if 75.7% of respondents knew about the Health District actions against malaria, few of them have been able to cite one specific intervention is currently conducted, such as rapid and free access to care (2.8%), bed-net (6.6%), house spraying (25.0%), provision of coils (2.4%) and educational training (6.0%). A wide majority of respondents (78.7%) declared being worried by malaria, but only 44.9% of them thought that malaria could kill, less than one of ten (28.3%) cited malaria as a major health problem for local population, ranged after diabetes and HIV/AIDS. We observed that 15.2% said having never heard anything about malaria before the investigation.

Many of the respondents reported night activities (85.9%). Among those nocturnal actives, 75.2% were back at home before 9:00 pm and were outside basically for socializing (77.8%). Fewer of them were outside for eating (20.2%), cooking (18.1%) or working (1.1%). When outside, respondents who were active at night reported specific preventive behaviours such as the use of spray (11.4%), fan (1.4%), coils (8.9%), fire (6.8%), specific beverage (1.1%) or an area cleaning (1.1%). It is noteworthy to mention than wearing long clothes at night was the most frequent preventive behaviours described (25.1%). A large proportion of respondents (49.4%) did not report any specific preventive behaviours during night-time activities. When inside, only 5.2% of respondents reported using specific measures to prevent malaria such as closing windows and doors. Focusing on sleeping behaviours, despite a good opinion toward bed nets shared by nearly all the respondents (90.6%), less than a quarter of them (22.9%) declared using it even though three quarters of the respondents were sleeping in a bed (75.4%). Among those who reported sleeping outside when too hot (59.6%), 77.0% did not use a bed net.

### Malaria history

Almost a third of respondents (31.9%) reported a malaria episode occurring during the previous 5 years, but in only 15.2% did the last episode occur from 2018 (as considered occurring during the previous year). Among them, 36.3% declared several episodes and almost all were at home for the last one (91.2%), had access to medical care (97.0%), and received treatment in the 3 days (55.2% during the two first days after symptoms). When comparing respondents that have reported having a malaria episode during the past years to others, no significant statistic differences appeared regarding social characteristics age, gender, education level or employment; including attitudes or knowledge measured or the night-time activities described and linked prevention behaviours.

By contrast, as shown in Table 3, living in Nkotswi was significantly associated with reporting a malaria episode as well as living in a household unit with the RPF profile. Concerning attitudes, respondents that did not consider housing conditions as a main living problem, were more prone to declare having a malaria episode. Concerning preventive behaviours, if a tendency differs respondents declaring outside activities at night to staying inside, those using spray declared significantly more often a malaria episode occurring during the past year. After multivariate adjustments (Table 3), determinants associated with a higher propensity among respondents to declare having a malaria episode during the past first years were: sex, profile of the household, location sleeping behaviours and an opinion on living conditions. Being a man, living in Nkotswi, living in a poor family and sleeping outside when too hot were factors positively associated to a reported past malaria episode. In the opposite way, paying attention to housing conditions stayed significantly associated to no malaria episode report.

## Discussion

This study used a community-based survey which was aimed at analyzing the association of individual behaviour and social organization factors with having at least one malaria episode and relate these findings to housing condition and health behaviour, among inhabitants in a malaria endemic area in the east Limpopo River Valley.

### Water proximity and quality

The proximity of water generated by rainfall, water courses or due to human activities could partly explain the housing risk level due to house locations. In addition to the impact of the proximity to water bodies on malaria exposure, ongoing growing number of publications involved other factors associated to the water type or quality [13] even in drought period [14]. At least, ecological changes also impacting the distribution of *Anopheles* mosquitoes should be considered especially considering new species within the area [15].

### Migration and seasonal workers: a pathway infection?

The results identifying a migrant/seasonal worker as a major conclusive social profile underlined the importance of investigating cross-border workers as a specific group with their own lifestyle habits that could mainly affect their health status [16]. This is consistent with health policy investigations aimed at targeting emerging health phenomena associated with invisible social networks in a cross-border context in order to call for more cooperative interventions at the international level [17]. Cross-border labor mobility, partly driven by economic conditions, is a major challenge for public health strategies, particularly in South Africa, where migrants and rural workers are already considered as a high-risk group for infectious diseases, including HIV [18]. A recent study has shown that the specific social characteristics of these hidden labor population groups and their associated mobility resulted in an epidemiological pattern generated by particular infection pathways called “corridors”[19]. In the context of malaria, migration remains a major problem for control strategies [20].

### Lessons from the results

Focusing on housing conditions and health behaviours, and migrant workers, the relationship between vulnerability to infection and poor housing conditions did not seem to be as obvious or specific as already reported in studies along the Amazon River [21, 22]. Housing interventions are mainly used to prevent malaria in any target population [23] and the fragility of frontier workers is more complex. The area method framing of the survey implied de facto the inclusion of many population groups and the comparative approach using a hierarchical selection of social profiles showed that deleterious housing conditions were not only associated with migration but also with the income level of sedentary families.

Mobility [24], especially cross-border mobility [25, 26], emerged as a more effective and endogenous determinant to characterize seasonal/migrant workers as a homogeneous population group radically different from others. Following the results obtained, the under-reporting of malaria episodes among migrants/seasonal workers, which remained significantly lower than among the poorest families, suggests that understanding malaria risk among them should be approached in two complementary and more specific ways using the above-mentioned “corridor” concept.

Focusing on the level of individual risk, exposure to malaria may be part of the self-vulnerability principle of members. Thus, mobility affecting routine practices and health behaviours generates a relapse into prevention. For example, bedding-related behaviours, such as access to a bed net or a good, quality room, become emblematic changes in health prevention behaviours that generate new infections [27].

Given the level of risk and vulnerability of the community, another perspective could be put forward to address the question of asymptomatic transport of the parasite by mobile and hidden groups. This is to identify the epidemic pathway related to human mobility that could partly explain the persistence of malaria in the particular area. More broadly, because of changes in mobility associated or not with climate change or economic crises, infectious pathways are geographical gateways for the emergence and spread of malaria or other infectious diseases. The “corridor” concept could help to adapt the survey to show what is happening in a hidden world and also to implement specific interventions.

### Fundamental causes or health behaviour?

In the last decade, social epidemiology, driven by the current craze for the theatrical theory of root causes developed by Link and Phellan, has strongly placed the social hierarchy caused by economic relations between groups at the center of the mechanism of health inequalities, which fully explains epidemic trends [28]. A critical point of view could be supported by the results of the study, which renew the interest in the relationship between external, social and collective constraints and individual freedom and subjectivity in health behaviours. The two-step analysis, carried out at the level of the household unit and the individual, allowed for the admittance of the effective and massive weight of socio-economic status on individual vulnerability to malaria, if one considers that the members of the household relatively grouped in the poorest profile declared to be the most exposed.

Moreover, combining the KAP data in a single survey allowed for being limited to a structural and economic view and not to exclude individual subjectivity. Despite the growing influence of the structural approach, from the 1980s onwards there was a large body of literature arguing for the effectiveness of health promotion intervention based on health education by assuming a direct relationship between health knowledge and health behaviour. The current critical position of researchers, supported by the persistence of the epidemic and risk behaviours in the population despite years and years of implementation of health education programmes and policies, cannot undermine all the successes already achieved and the evidence for this type of action [29, 30].

However, this success of health education does not exclude, as this study showed, that the risk behaviours that remain in the group and population regularly studied and trained are not negligible, for example sleeping behaviour or the use of prevention during the night. Other explanations could be discussed, including inter-individual or community dimensions. Ongoing social interactions in the local cultural context should also be considered as potential factors directly or indirectly influencing individual health behaviour. Community beliefs, which were not assessed in this survey, need to be further investigated in order to identify potential causes for the persistence of risk behaviours.

Indeed, comprehensive interventions to reduce the incidence of malaria in the general population should not rely exclusively on one approach. In addition to targeted health promotion programmes, actions at an intermediate level, such as social networks and communities, as well as at a broader level focusing on the economic well-being of society and the quality of life of the population should be pursued with a view to achieving complete eradication of malaria. However, a published paper promotes another possible way forward based on stricter global control [31]. The balance between human rights and individual freedom on the one hand, and centralized, strict control on the other, introduces a new aspect of weighing up the pros and cons of interventions, without forgetting the central concern along the way in choosing to change people's lifestyles, or more simply to enable them to reduce risk without the ambition to change their lifestyles.

### Gendered differences in life style, behaviours and powered relationship

Other things being equal, a significant independent association between gender and malaria showed that men were more exposed than women. In contrast to previous report that women are more exposed than men [32], the findings may be attributed to men in the study area being frequent travelers due to border migration for work, increasing their exposure. However, social desirability bias and possible under recruitment of men during sampling cannot be excluded. Furthermore, apart from these exceptions, other types of explanation can be put forward and highlight the role played by masculinity in risky health behaviours from the perspective of the social construction of gendered identity over the course of a lifetime [33], assimilating male attitudes and stereotypes from the first stage of the construction of the self [34]. Recently, gendered health behaviours were observed during the COVID-19 pandemic showing that men in many countries were less likely to wear a mask in public [35]. More specifically, with regard to malaria transmission, it has been documented for years that in rural areas, men's lifestyles, especially night-time activities, contribute to deleterious health behaviours and increase exposure to the risk of becoming infected [36].

### Limits and strengths

In this study, collection of data was based on a day-time investigation, during working hours, which was a limitation as it drastically reduced the availability of part of the population and generated an imbalance on gender participation to the survey. Additionally, malaria history was only collected through reported experiences. Lastly, the lack of official data on local population makes it difficult to define quality of the sample in terms of representability. Conversely, originality of this survey provided updated information on a vulnerable South African population towards malaria still less surveyed, indeed unknown.

Despites these limitations, significant differences of impact of malaria based on respondent’s reports highlight the importance of global life conditions including location of housing, mobility related to working status, and economic level linked to housing conditions. The findings do not exclude individual characteristics as possible cause of a higher exposure to malaria. Results of multivariate modeling designated also subjectivity through health behaviours or gender. Furthermore, the design, which combines in one analysis two distinguished levels of factors, yielded a more complete interpretation of linkage between individual behaviours and surrounding context.

## Conclusion

The malaria elimination agenda is a high priority for malaria endemic countries such as South Africa and the United Nations Sustainable Development Goals (specifically SDG 3.3). Thus, in context of endemic malaria, the findings of this paper suggest the importance of considering social and contextual factors in assessing malaria risk. In addressing malaria elimination in South Africa and accounting for the level of vulnerability and risk of the community, considering a potential ‘corridor’ could be an approach to inform targeted malaria interventions and studying asymptomatic carriage in migrant workers in malaria endemic areas. Using the Fundamental Causes Theory, this study proposes that these interventions should include the integration of malaria control policies and health behaviour prevention to reinforce the malaria elimination agenda.

## Data Availability

The datasets generated during and/or analyzed during the current study are available from the corresponding author on reasonable request.

## Code availability

The codes are available from the corresponding author on reasonable request.
